# Development and Comparison of a Panel of Modified CS17 Fimbrial Tip Adhesin Proteins as Components for an Adhesin-Based Vaccine against Enterotoxigenic *Escherichia coli*

**DOI:** 10.3390/microorganisms9081646

**Published:** 2021-07-31

**Authors:** Yang Liu, Milton Maciel, Aisling O’Dowd, Steven T. Poole, Julianne E. Rollenhagen, Irina V. Etobayeva, Stephen J. Savarino

**Affiliations:** 1 Henry M. Jackson Foundation for the Advancement of Military Medicine, Bethesda, MD 20817, USA; mmacielnmrc@gmail.com (M.M.J.); ashodowd@hotmail.com (A.O.); spoole102@gmail.com (S.T.P.); julianne.e.rollenhagen.ctr@mail.mil (J.E.R.); 2 Enteric Diseases Department, Naval Medical Research Center, Silver Spring, MD 20910, USA; irina.v.etobayeva.mil@mail.mil (I.V.E.); s.j.savarino@gmail.com (S.J.S.)

**Keywords:** CS17 ETEC, CsbD, vaccine, prophylactic, allele matching, protein engineering

## Abstract

Enterotoxigenic *Escherichia coli* (ETEC) is a leading cause of diarrhea in travelers and children in resource-limited countries. ETEC colonization factors, fimbrial tip adhesins and enterotoxins are key virulence factors, and thus have been studied as vaccine candidates. Some prevalent colonization factors, including CFA/I and CS17, belong to the class 5 family. We previously found that passive oral administration of hyperimmune bovine colostral IgG (bIgG) raised against dscCfaE (donor strand complemented CFA/I tip adhesin) protected volunteers against CFA/I^+^ ETEC challenge, while anti-dscCsbD bIgG (CS17 tip adhesin) did not confer protection. These findings led us to develop and optimize a panel of alternative CsbD-based vaccine candidates based on allele matching and in silico protein engineering. Physicochemical characterizations revealed that an optimized vaccine candidate dscCsbD_LSN139_(P218A/G3) had the greatest thermal stability among the six tested dscCsbD adhesins, whereas the overall secondary structures and solubility of these adhesins had no obvious differences. Importantly, dscCsbD_LSN139_(P218A/G3) elicited significantly higher CS17^+^ ETEC hemagglutination inhibition titers in sera from mice intranasally immunized with the panel of dscCsbD adhesins, while no significant difference was observed among heterologous neutralizing titers. Our results strongly advocate for the incorporation of these modifications into a new generation of CsbD-based ETEC vaccine candidates.

## 1. Introduction

Enterotoxigenic *Escherichia coli* (ETEC) has been identified as one of the top five pathogens causing diarrhea among children under age five in regions such as South Asia and Sub-Saharan Africa [1]. Further, ETEC has remained the leading etiology of travelers’ diarrhea among adults visiting low-to-middle-income countries [2,3]. ETEC is estimated to cause over 50,000 deaths and several hundred million of diarrheal cases per year [4]; the dismal situation is exacerbated by rising antibiotic resistance [5] and no licensed ETEC vaccine. A few current ETEC vaccine candidates in clinical trials pivot on ETEC colonization factors, fimbrial tip adhesins and enterotoxin toxoids, as they are recognized as protective antigens. However, more than 25 ETEC colonization factors displaying distinct serotype diversity have been identified on the surface of clinical ETEC isolates, which complicates the vaccine development. Class 5 fimbriae, CS3, CS6, and CS21 are predominant ETEC colonization factors significantly associated with moderate to severe diarrhea [3,6].

The class 5 fimbriae have eight members, which are split into 5a (CFA/I, CS4, CS14), 5b (CS1, CS17, CS19, PCFO71) and 5c (CS2) subclasses [7]. CFA/I, the prototype class 5 fimbriae, has long been established as an ETEC virulence factor and a protective antigen [8,9]. CfaE, the CFA/I fimbrial tip adhesin, is the functional adhesive subunit of CFA/I [10,11,12]. Importantly, CfaE serving as a vaccine antigen, or prophylactics targeting CfaE, have been shown to be effective in reducing CFA/I-expressing ETEC colonization in the mouse model and ETEC-induced diarrhea in the non-human primate [13,14,15,16]. In a randomized clinical trial, hyperimmune bovine colostrum specific to CfaE or CFA/I granted protective efficacy when human volunteers were challenged with homologous CFA/I^+^ ETEC strain H10407 [17]. The findings of this study motivated a second trial with a focus on CS17^+^ ETEC. In this second study, a design similar to the first study was implemented in which hyperimmune bovine colostrum raised against CS17 or CsbD (CS17 fimbrial tip adhesin) was prepared and investigated in a controlled human infection model [18]. The bovine colostrum specific to CS17 protected the volunteers, while the colostrum specific to CsbD, the CS17 fimbrial tip adhesin, failed to show protective efficacy when volunteers were challenged with a CS17^+^ ETEC strain. A few considerations of the divergent findings in the two studies were contemplated, including the purification method, as well as the excipient and allelic variation of the CsbD immunogen used to generate the bovine colostrum [18].

In the current study, we optimized the CsbD-based vaccine candidates based on allele matching and protein engineering, characterized six antigens in physiochemical assays, and demonstrated improved serum responses in mice immunized with the modified antigens.

## 2. Materials and Methods

### 2.1. CS17 Vaccine Antigen Design by Allele Matching and Structural Modelling

CsbD_LSN139_ from a prevalent ETEC strain LSN02-013966/A (hereafter as LSN139) was selected as a primary CsbD allele based on the previously published CS17 phylogenetic analysis [19]. In the hemagglutination inhibition (HAI) and ELISA assays described below, the CS17^+^ ETEC strain and CS17 fimbriae were matched to this primary CsbD allele. To produce structural models of dscCsbD_LSN139_ and its variants, the protein sequences and a structural template from dscCfaE crystal structure (PDB ID: 2HB0) were used as input in MODELLER [20]. The structural models of dscCsbD_LSN139_ and its variants were energy minimized and examined. The structure figures were prepared in UCSF Chimera [21].

### 2.2. Molecular Cloning and Protein Purification

The resolubilized donor strand complemented CsbD from strain WS6788 (hereafter as dscCsbD_WS6788_) was previously cloned and purified [18]. The dscCsbD_LSN139_ was constructed similarly by connecting the C-terminus of CsbD sequence from the ETEC strain LSN139 to the donor strand of VEKNITVRASVDPKLDLLQ with a tetrapeptide linker DNKQ. The dscCsbD_LSN139_ was used as a template to clone the following mutants. A point mutation of P218A in dscCsbD_LSN139_ (hereafter as dscCsbD_LSN139_(P218A)), a tripeptide linker GGG replacing the tetrapeptide linker DNKQ in dscCsbD_LSN139_ (hereafter as dscCsbD_LSN139_(G3)) and combination of P218A and G3 mutations in dscCsbD_LSN139_ (hereafter as dscCsbD_LSN139_(P218A/G3)) were made using QuikChange (Agilent, Santa Clara, CA, USA) site-directed mutagenesis. The resulting plasmids were transformed into BL21(DE3) cells (Novagen, Danvers, MA, USA). The bacterial cells were grown in APS media at 32 °C until the OD reached 0.6. The bacterial culture was then induced with IPTG at a final concentration of 1 mM. The cells were harvested by centrifugation after 3 h induction at 32 °C. The soluble dscCsbD_WS6788_, dscCsbD_LSN139_, dscCsbD_LSN139_(P218A), dscCsbD_LSN139_(G3) and dscCsbD_LSN139_(P218A/G3) were purified in a similar way. Briefly, the cell paste was resuspended in PBS (1× *g* of cell paste in 5 mL of PBS). The suspension was passed through a microfluidizer twice, and the cell lysate was centrifuged at 10,000 rpm for 40 min at 4 °C. The supernatant was loaded into a 5 mL HisTrap column (Cytiva, Marlborough, MA, USA) at a flow rate of 5 mL/minute using AKTA Explorer. After loading, the column was washed with 5–10 column volumes (CV) of a buffer containing 20 mM phosphate, 0.5 M sodium chloride and 5 mM imidazole, pH 7.4. The protein was eluted with a buffer containing PBS and 200 mM imidazole, pH 7.4. The eluates from the HisTrap column were diluted 20 times with a buffer containing 20 mM MES, pH 5.5 (buffer A), and loaded on a HiTrap SP HP column (Cytiva, Marlborough, MA, USA). The eluates from the SP column were eluted using a linear gradient from the buffer A to a buffer containing 20 mM MES, 500 mM sodium chloride, pH 5.5 over 30 CV. The fractions containing the target protein were pooled and concentrated.

### 2.3. Physiochemical Characterization of Proteins

The denatured and reduced CS17 adhesins and variants were examined on 15% SDS-PAGE gels and the protein purity was evaluated by densitometry. The endotoxin level in each protein was determined by LAL gel clot assay (Associates of Cape Cod, MA, USA). The solubility of the proteins was measured as follows. The protein solution (concentration and buffer) was lyophilized and resuspended in water with a volume eight times less than the original volume of protein solution. The resuspended solution was visually examined and centrifuged at 16,000× *g* for 5 min. After centrifugation, the supernatant concentration was determined by the BCA assay (Pierce, ThermoFisher, Waltham, MA, USA). The secondary structure and melting temperature of each protein was measured by circular dichroism (CD). Briefly, each adhesin at 0.1 mg/mL in a 1 mm cuvette was scanned in a Jasco 810 circular dichroism spectrometer (Jasco, Great Dunmow, Essex, UK) from 250 nm to 190 nm at a rate of 10 nm/min. Ellipticity values of three scans at 20 °C were averaged, and this average was used to estimate the secondary structure. The melting temperature was taken as the middle point of the spectra during thermal transition when the temperature was ramping up from 20 °C to 95 °C and the wavelength was fixed at 230 nm.

### 2.4. Murine Immunizations

Animal studies were approved by the Institutional Animal Care and Use Committee at the Naval Medical Research Center in compliance with all applicable federal regulations governing the protection of animals and research. Female BALB/c mice, ages 6–8 weeks (Jackson Laboratories, Bar Harbor, ME) were vaccinated by the intranasal (IN) route with the CS17-based antigens listed in Table 1 in a three-dose schedule on days 0, 14 and 28. Antigens were administered to groups of 9–10 mice each in sterile PBS with LT(R192G). Antigens and LT(R192G) were given in 25 µg and 1.5 µg doses, respectively. One group of animals received PBS alone. Vaccinations were carried out as previously described [22].

### 2.5. Immunological Sampling and Measurement of Immune Response

Blood samples were collected via cardiac puncture on day 42. Whole blood was centrifuged at 400× *g* at 4 °C for 15 min and the separated serum was stored at −20 °C. To detect IgG and IgA antibody responses to dscCsbD, microtiter plates were coated in a humidified chamber at 37 °C for 1 h and subsequently overnight at 4 °C (IgG) or in a humidified chamber at 37 °C overnight (IgA) with 50 ng (IgG) or 200 ng (IgA) of antigen per well in 1X PBS (Sigma–Aldrich, St. Louis, MO, USA). Plates were washed with PBS, then blocked for 30 min at 37 °C with 1% fetal calf serum (FCS; Serologicals, Atlanta, GA, USA) in 1X PBS 0.05% Tween 20 (PBS-T; Sigma–Aldrich) (IgG) or for 1 h at 37 °C with 5.0% nonfat milk (Sigma–Aldrich) in 1X PBS-T (IgA). Plates were washed with PBS-T and sera were added in three-fold serial dilutions, beginning with a dilution of 1:50 in 0.1% FCS in 1X PBS-T (IgG) or 1.0% nonfat milk in 1X PBS-T (IgA). Plates were then incubated at room temperature (RT) for 90 min. Plates were washed with PBS-T and goat anti-mouse IgG HRP conjugate (Jackson ImmunoResearch Laboratories, Inc., West Grove, PA, USA) was applied to each well at a 1:1000 dilution in 0.1% FCS in 1X PBST (IgG) or biotin-labeled goat anti-mouse IgA secondary antibody (KPL, Milford, MA, USA) was applied to each well at a 1:1000 dilution in 1.0% nonfat milk-1X PBST (IgA). Plates were incubated at RT for 90 min, washed with PBS-T. For IgG assays, plates were developed with orthophenylenediamine (Sigma–Aldrich) as per the manufacturer’s instructions and read after 20 min. Alternatively, for IgA assays, extravidin–peroxidase (Pierce, Waltham, MA, USA) diluted 1:1500 in 1.0% FCS in 1X PBST was added to each well and the plates were incubated in a humidified chamber for 30 min at RT. Plates were then washed with 1X PBST, 1-Step Ultra TMB (Pierce) was added and then plates were incubated for 30 min at RT. TMB Stop Solution (KPL) was added to each plate, and plates were incubated for 2 min at RT. For both IgG and IgA assays, the optical density at 450 nm was determined with a Multiskan Ascent ELISA plate reader (Thermo Fisher Scientific, Waltham, MA, USA). Endpoint titers were calculated as the reciprocal of the interpolated dilution giving an A_450_ of 0.4 units above background. Antibody titers ascribed to each sample represents the mean of duplicate samples, run on consecutive days. Samples with a reciprocal end-point titer of <50, the lowest dilution tested, were assigned a value of 25 for computational and graphical purposes.

A hemagglutination inhibition (HAI) assay was used as the proxy for neutralization of fimbriae-mediated adhesion and performed on sera from study day 42. Serum samples were tested in 96-well plates (Falcon Microtest™ U-bottom tissue culture treated, BD, Franklin Lakes, NJ, USA) as follows. Each day, the minimum hemagglutination titer (MHT) was determined, defined as the reciprocal of the highest dilution of bacteria that agglutinated bovine erythrocytes (Lampire Biological Laboratories, Pipersville, PA, USA). CS17^+^ ETEC strain LSN139, CS1^+^ ETEC strain WS1974A, CS19^+^ ETEC strain WS0115A and PCFO71^+^ ETEC strain WS2173A were grown overnight on CFA agar plates, harvested, and resuspended in 0.5% D-mannose (Thermo Fisher Scientific) in PBS (PBS-M) to a final solution with OD_650_ of 20 ± 2.0. Twenty-five µL of this suspension was added to the wells followed by 2-fold serial dilution with PBS-M. Twenty-five µL of 1.5% erythrocytes in PBS-M was added to each dilution, followed by 25 µL of PBS-M for a total of 75 µL per well. Plates were agitated at 500 RPM for 30 min at 4 °C. The highest dilution of bacteria to give visible agglutination was taken as the MHT for the day. The HAI assay was performed with a bacteria suspension two dilutions more concentrated than the MHT. For HAI assays, 25 µL of 2-fold serial dilutions of serum in PBS-M, beginning with 1:8 was added to wells with equal volume of bacterial suspension, forming final serum dilutions beginning with1:16. Plates were agitated for 30 min at RT and then 25 µL of 1.5% erythrocytes was added to each well and plates were agitated at 500 RPM for 30 min at 4 °C. The presence or absence of agglutination was recorded immediately after incubation. Each sample was tested in duplicate and the HAI titer was defined as the average of the reciprocal of the highest serum dilution that completely inhibited agglutination. Serum samples that did not agglutinate at the lowest dilutions tested were assigned reciprocal titers of 8, for computational purposes.

### 2.6. Statistical Analysis

All statistical analysis and graphing were performed using GraphPad Prism Version 9.1.0 for macOS (Graphpad Software Inc., San Diego, CA, USA). Serum ELISA and HAI titers were normalized by a log_10_ transformation prior to statistical analysis. Group titers were compared using a one-way analysis of variance (ANOVA) and a Tukey’s *post hoc* test was used for pair-wise comparisons. For all statistical comparisons, *p* < 0.05 was regarded as significant and tests were interpreted in a two-tailed fashion.

## 3. Results

### 3.1. Antigen Allele Matching and in Silico Engineering

The CS17 adhesin CsbD has been reported to have seven allelic variants in amino acid sequences [19], and allele matching of vaccine antigens plays a significant role in eliciting protective responses [23,24]. Based on the phylogenetic analysis of 18 CS17-expressing ETEC strains, the LSN139 allele of CsbD is a highly prevalent allele [4,19]. Therefore, the CsbD/LSN139 allele was adopted in designing new adhesin-based subunit vaccine candidates for CS17^+^ ETEC. The *in cis* donor strand complementation (dsc) strategy previously used in designing dscCfaE [11] was retained to stabilize the CsbD antigen (dscCsbD_LSN139_), whereby the N-terminal 19 residues from mature CsbA (the major subunit which forms the stalk of the fimbriae) were connected to the C-terminal CsbD using a tetrapeptide linker DNKQ.

**Figure 1:**
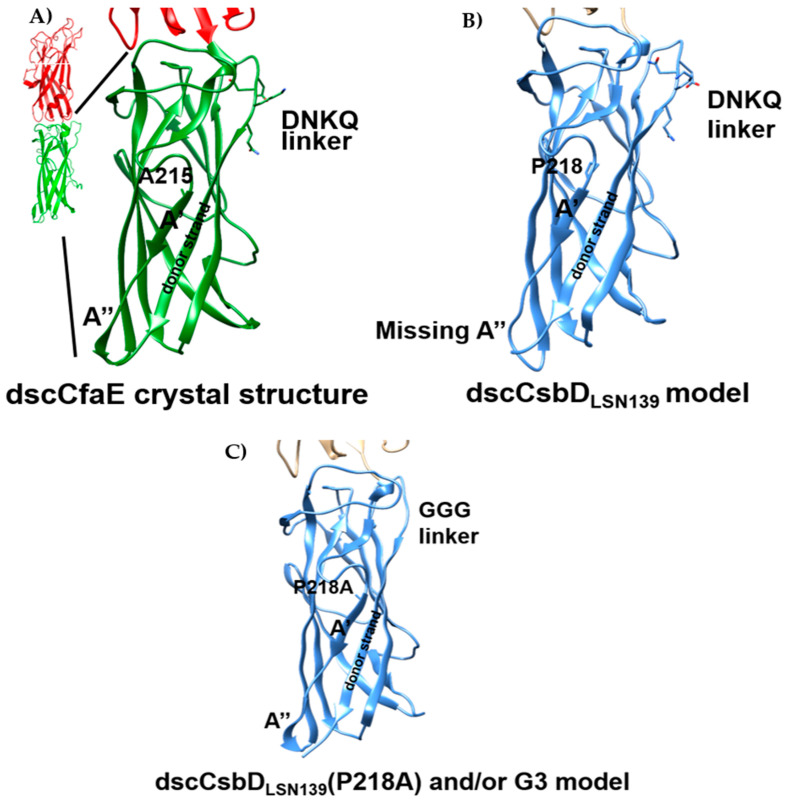
dscCfaE crystal structure and dscCsbD_LSN139_ structural models. (**A**) A’ strand, A’’ strand, DNKQ linker, alanine 215 and the donor strand were marked in the pilin domain of dscCfaE crystal structure (PDB ID: 2HB0). (**B**) A’ strand, the missing A’’ strand, DNKQ linker, proline 218 and the donor strand were marked in the pilin domain of dscCsbD_LSN139_ structural model. (**C**) A’ strand, the recovered A’’ strand, GGG linker, point mutation P218A and the donor strand were marked in the pilin domain of dscCsbD_LSN139_(P218A) and/or G3 structural model.

Additionally, the structural integrity of a subunit vaccine antigen may contribute to vaccine solubility, oligomerization state, thermostability, immunogenicity and eventual protective efficacy [25]. Therefore, we modeled the three-dimensional structure of dscCsbD_LSN139_ in silico using the crystal structure of dscCfaE as a template (Figure 1A). The overall structural model of dscCsbD_LSN139_ resembled that of dscCfaE with adjoining adhesin and pilin domains, and each domain consisted of seven β-strands forming two layers of a β-sandwich. However, the A” strand in the pilin domain was disordered in the structural model of dscCsbD_LSN139_ (Figure 1B) when compared to the same region in the dscCfaE structure. Since the A’ and A” strands are adjacent to the donor strands in both structures, and important for maintaining the structural integrity of the vaccine antigens, remedies were sought to recover the A” β strand. In silico analysis of the structural model revealed that either mutation of the proline at position 218 to an alanine dscCsbD_LSN139_(P218A) or replacing the tetrapeptide DNKQ linker with a tripeptide GGG linker dscCsbD_LSN139_(G3) resulted in the reappearance of the A” strand (Figure 1C). A combination of P218A mutation and G3 linker in dscCsbD also resulted in recovery of the A” β strand based on the structural model.

Based on the allele matching and analysis of the structural model, dscCsbD_LSN139_, dscCsbD_LSN139_(P218A), dscCsbD_LSN139_(G3) and dscCsbD_LSN139_(P218A/G3) were engineered and cloned using the *csbD* nucleotide sequence from the CS17 ETEC LSN139 allele.

### 3.2. Physiochemical Characterization of Vaccine Antigens

We purified dscCsbD_LSN139_, dscCsbD_LSN139_(P218A), dscCsbD_LSN139_(G3), dscCsbD_LSN139_(P218A/G3) and dscCsbD_WS6788_ from soluble fractions of the cell lysate, and characterized them in terms of physiochemical properties, together with resolubilized dscCsbD_WS6788_, which was made previously from the insoluble fractions [18]. Since the resolubilized dscCsbD_WS6788_ was prone to aggregate and precipitate, certain characterization assessments were not performed on this antigen. The purity, endotoxin levels, solubility, size-exclusion chromatography profile, secondary structure and melting temperatures of the proteins are summarized in Table 1. The six dscCsbD antigens were highly purified and had low endotoxin levels. After resuspending the lyophilate of the tested antigens in water, the resuspension was centrifuged, and no precipitation was observed. The concentrations of the supernatant from the reconstitution of the dscCsbD lyophilate were determined at 8.0 mg/mL, which suggested the solubility of the tested dscCsbD was equal to or greater than 8.0 mg/mL. The dscCsbD antigens were mainly monodisperse according to the results from the size-exclusion HPLC. Based on the circular dichroism spectra, the secondary structures were mainly β sheets, which is consistent with the structural modeling (Figure 1B,C). There was no difference in melting temperatures (Tm) between soluble dscCsbD_WS6788_ and dscCsbD_LSN139_. The Tm of dscCsbD_LSN139_(P218A) mutant decreased by 1 °C compared to the Tm of wild type dscCsbD_LSN139_, which was unexpected as we anticipated the P218A mutation would recover the A’’ β strand and increase the thermostability. However, the Tm of dscCsbD_LSN139_(G3) mutant increased by 2 °C compared to that of the wild type dscCsbD_LSN139_, and the Tm of dscCsbD_LSN139_(P218A/G3) mutant increased by 3 °C compared to that of the dscCsbD_LSN139_(P218A), suggesting that the G3 linker played the dominant role in increasing the thermostability of the dscCsbD antigens.

### 3.3. Immunogenicity and Functional Antibody Responses

**Figure 2:**
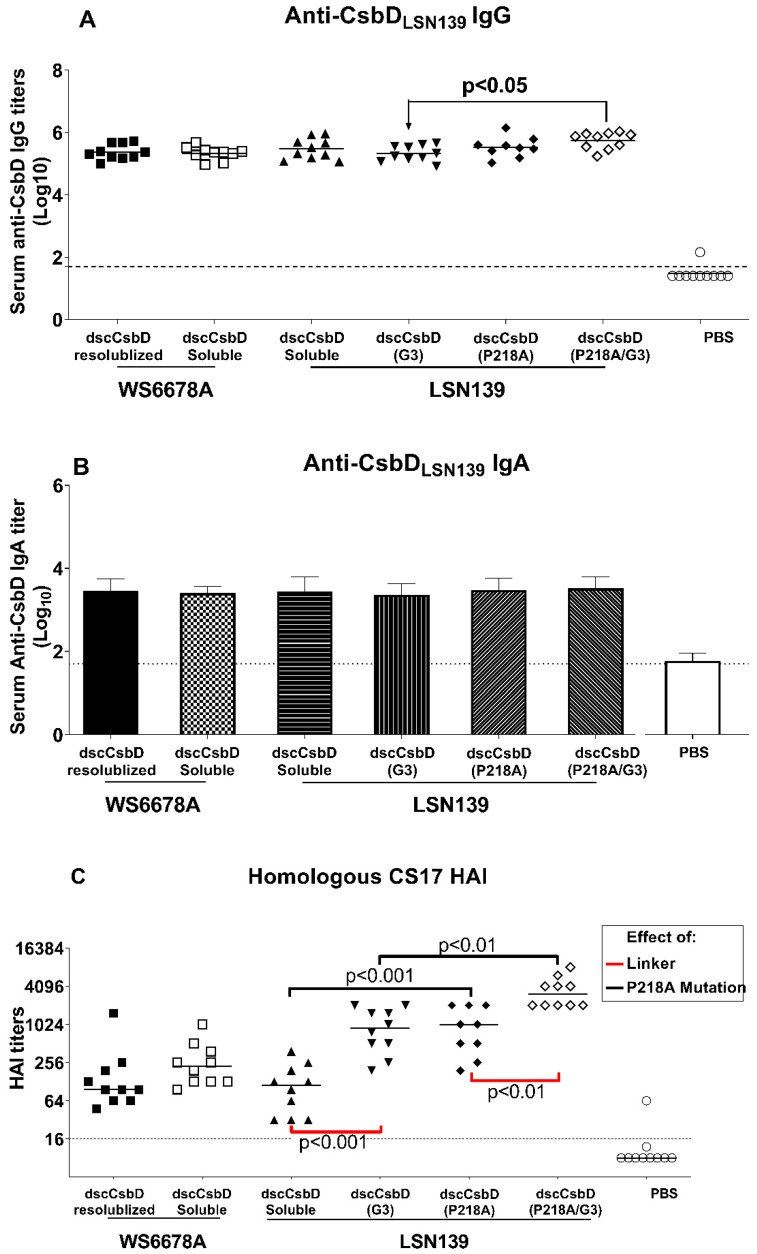
Serum antibody responses in mice immunized IN with CsbD-based antigens. Mice (9–10/group) were immunized on days 0, 14, and 28 with 25 µg of antigen and with 1.5 µg LT(R192G). (**A**) Serum anti-dscCsbD_LSN139_ IgG antibody responses on day 42. (**B**) Serum anti-dscCsbD_LSN139_ IgA antibody responses on day 42. (**A**,**B**) All values shown are the individual log_10_ titers in serum collected two weeks after the third dose (day 42). Solid horizontal lines represent the mean of each group, and the horizontal dotted lines denote the lowest dilution tested (1:50). (**C**) Functional antibody titers measured by the HAI assay using CS17^+^ ETEC strain LSN139. Individual values are shown with the medians of each group represented by a horizontal solid line. Horizontal dotted lines denote the lowest dilution tested for the assays (1:16). Statistical significance was determined by using a one-way ANOVA and Tukey’s post hoc test.

**Figure 3:**
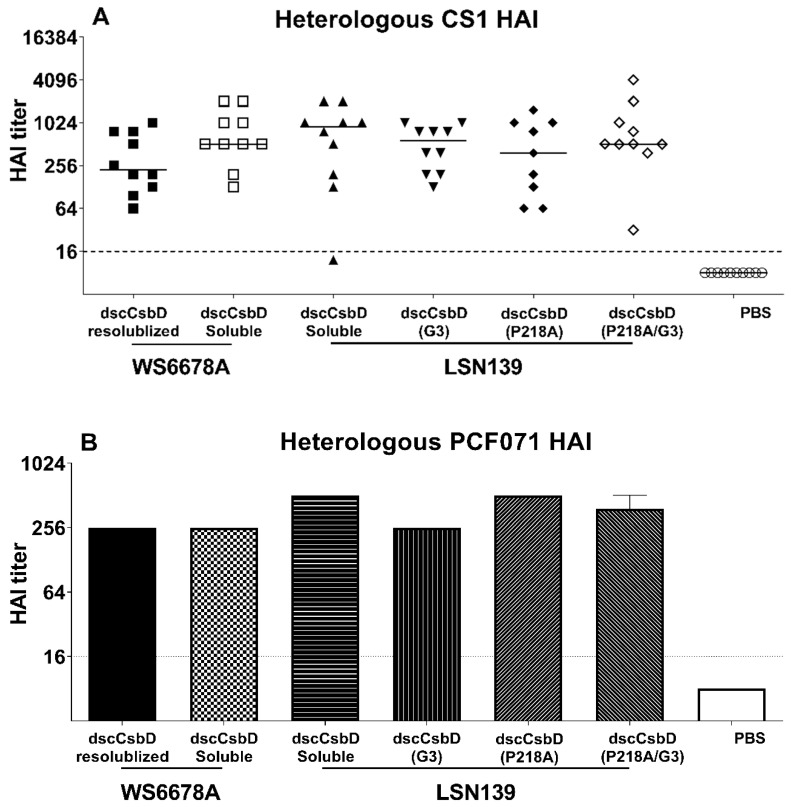

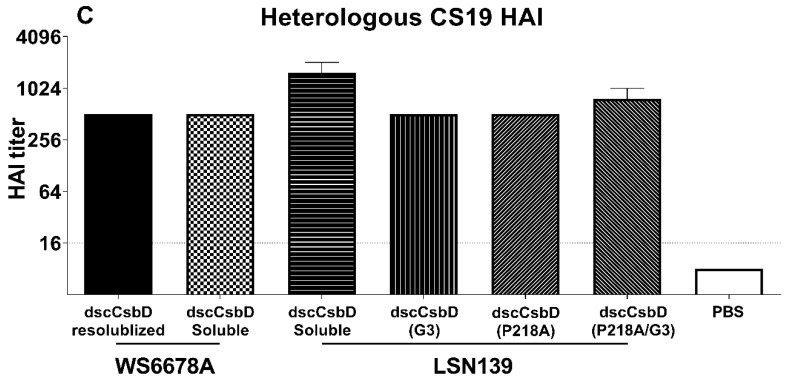
Functional antibody titers measured by the HAI assay using heterologous class 5b ETEC strains (**A**) CS1^+^ ETEC strain WS1974A, (**B**) PCF071^+^ ETEC strain WS2173A, (**C**) CS19^+^ ETEC strain WS0115A. (**A**) Individual values are shown with the median of each group represented by a horizontal solid line. (**B**,**C**) HAI titers of pooled samples are shown as bars. Horizontal dotted lines denote the lowest dilution tested for the assays (1:16).

Mice were intranasally immunized with 25 µg of each dscCsbD antigen plus 1.5 µg LTR192G on days 0, 14, and 28. Sera collected on day 42 were evaluated in anti-CsbD ELISA (IgG and IgA) and HAI assays (Figure 2 and Figure 3). In the anti-dscCsbD_LSN139_ IgG ELISA assay, all antigens elicited high levels of anti-dscCsbD_LSN139_ IgG antibodies. The combination of P218A mutation and GGG linker in dscCsbD_LSN139_(P218A/G3) elicited significantly higher titers than dscCsbD_LSN139_(G3) (Figure 2A, black horizontal bars).

In the anti-dscCsbD_LSN139_ IgA ELISA assay (Figure 2B), there was no statistical difference in titers elicited by various dscCsbD antigens.

The ability of the CsbD-based antigens to induce functional neutralizing antibody titers using the HAI assay was assessed. Using the homologous CS17^+^ ETEC LSN139 strain, the effects of allele matching, linker and P218A mutation were specifically investigated. The inclusion of the GGG linker significantly boosted HAI titers in dscCsbD_LSN139_(G3) and dscCsbD_LSN139_(P218A/G3) compared to dscCsbD_LSN139_ and dscCsbD_LSN139_(P218A), respectively (Figure 2C, red brackets). The P218A mutation also led to significantly increased HAI titers in dscCsbD_LSN139_(P218A) and dscCsbD_LSN139_(P218A/G3) compared to dscCsbD_LSN139_ and dscCsbD_LSN139_(G3), respectively (Figure 2C, black brackets). Of note, the median HAI titers induced by dscCsbD_LSN139_(P218A/G3) was 27-fold higher than the median HAI titers induced by unmodified dscCsbD_LSN139_.

Additionally, the heterologous functional serum HAI titers induced by dscCsbD antigens were evaluated using three other class 5b ETEC strains. All dscCsbD antigens elicited heterologous HAI responses against the CS1, CS19 and PCFO71-expressing ETEC strains (Figure 3A–C); however, no significant difference was observed in the heterologous HAI titers using CS1 ETEC strain WS1974 among the dscCsbD antigens. HAI assays using CS19 and PCFO71 strains WS2173A and WS0115A, respectively, were run on pooled sera and therefore could not be analyzed statistically. Overall, the heterologous HAI responses were generally lower than the observed homologous HAI responses.

## 4. Discussion

ETEC is a major etiology of morbidity and mortality caused by bacterial diarrhea in travelers and pediatric population in endemic regions [1,3]. No ETEC specific vaccines have been licensed. Current ETEC vaccine candidates being tested in human clinical trials target prevalent ETEC CFs, fimbrial tip adhesins and enterotoxins [6], and new ETEC virulent factors have been identified as promising vaccine candidates as well [26,27]. CS17 is one of the predominant CFs from ETEC isolates in certain regions [3]. In the experiments presented herein, we set out to optimize the CsbD-based vaccine candidate that was previously used to produce dscCsbD_WS6788_-specific hyperimmune bovine colostrum, which was found to be inadequate in protecting against CS17^+^ ETEC challenge [18].

One consideration was that the recombinant dscCsbD_WS6788_ previously used was prepared from an insoluble fraction of cell lysate, denatured by urea, refolded during purification, and maintained in an imidazole-containing buffer, which may have resulted in a partially folded antigen or critical neutralizing epitopes being masked by excipients. In the current study, the original dscCsbD_WS6788_ expression clone and growth conditions were used to generate cell paste. The soluble dscCsbD_WS6788_ was then purified from supernatant of the cell lysate and released in a buffer without imidazole. The circular dichroism spectra of the resolubilized and soluble dscCsbD_WS6788_ suggested both proteins have a main secondary structure as beta sheets (Figure S1). No significant difference in anti-dscCsbD_LSN139_ IgG, IgA ELISA titers or HAI titers was observed in sera from mice immunized with the two proteins. A second consideration was that CsbD from WS6788 strain differs from the CsbD from strain LSN03-016011/A by three residues at residues 62, 85, and 144 [18,19]. Allelic mismatch between the vaccine immunogen and the challenge strain may have led to reduced or lack of efficacy as exemplified vaccine antigens for *Neisseria Meningitidis* serogroup B, where decreased serum bactericidal titers were induced by the factor H binding protein (fHbp, also known as LP2086) from subfamily B versus strains from subfamily A of *Neisseria Meningitidis* B [24]. According to the phylogenetic analysis [19], LSN139 CsbD allele was considered to be the most prevalent CsbD allotype and differs from WS6788 CsbD allele at residues 85 and 144. We cloned, purified and characterized the dscCsbD_LSN139_, and did not discover major differences in the physiochemical properties between soluble dscCsbD_WS6788_ and dscCsbD_LSN139_. In the mouse study, neither anti-dscCsbD_LSN139_ IgG, IgA ELISA titers nor homologous HAI titers elicited by the two antigens was significantly different.

Nevertheless, the in silico structural modeling results suggested that the P218A amino acid change and/or G3 linker could recover the A” strand secondary structure, so we introduced the two modifications in dscCsbD_LSN139_ context. The dscCsbD_LSN139_(G3) and dscCsbD_LSN139_(P218A/G3) proteins had melting temperatures 2 degrees higher than dscCsbD_LSN139_, which suggests that the two modified proteins are more thermally stable and is consistent with the in-silico modeling. Additionally, the sera from mice immunized with the dscCsbD_LSN139_(P218A/G3) had significantly higher anti-dscCsbD_LSN139_ IgG titer than those immunized with dscCsbD_LSN139_(G3). More importantly, the dscCsbD_LSN139_(P218A/G3) induced the highest homologous HAI titer among all dscCsbD proteins (Figure 2C). It is intriguing that P218A and/or G3 modifications promoting stability in the pilin domain of CsbD boosted the homologous neutralizing (HAI) titer because the receptor binding domain of CsbD and other class 5 fimbrial tip adhesins resides in the adhesin domain. One possible explanation is that a more stable protein will be less prone to degrade and last longer in vivo in order to elicit immune responses, which is supported by the fact that the dscCsbD_LSN139_(P218A/G3) had the highest anti-dscCsbD_LSN139_ IgG titer among all dscCsbD proteins (Figure 2A). A second hypothesis is that the receptor binding region in the CsbD adhesin domain is allosterically regulated by its pilin domain. More data exists for CfaE, a prototype class 5 fimbrial tip adhesin, to support the allosteric regulation. A CfaE/G168D mutant was found to disrupt the interface of adhesin domain and pilin domain, leading to structural reorganization in the pilin domain, which in turn affected the dynamic host cell binding property in the adhesin domain [28]. Additionally, we observed that individual monoclonal antibodies (mAbs) specific to the pilin domain of dscCfaE, dscCsbD or dscCotD, had hemagglutination inhibition activity against homologous ETEC strains [29]. The possible inhibiting mechanism may be that those mAbs bind and lock the pilin domain in a certain conformation and prevent allosteric activation of the receptor binding domain in the adhesin domain.

Although all dscCsbD antigens generated functional titers against other three (CS1, CS19, PCFO71) class 5b strains (Figure 3A–C), the heterologous HAI titers were not as high as the homologous HAI titers. To align with the goal of producing a potent multivalent ETEC vaccine with limited number of antigens, we are actively working to improve the coverage and potency of each antigen. It has been shown that hyperimmune bovine colostrum specific to CFA/I or CS17 conferred protective efficacy when human volunteers were challenged with CFA/I^+^ or CS17^+^ ETEC strains, respectively [9,17,18], and the class 5 ETEC fimbriae are constructed predominantly of the major subunits [30]. Therefore, we have previously engineered dscCfaEB, a fusion protein of the CFA/I minor subunit CfaE and the major subunit CfaB. The dscCfaEB was found to protect against diarrhea induced by a homologous CFA/I ETEC strain [31] and heterologous CS14 ETEC strain [32] in the non-human primate model. A similar fusion design can be applied to dscCsbD, in which one or a few major subunits from four class 5b fimbriae are tandemly linked to the C-terminal of CsbD.

In summary, data presented here provide evidence that allelic matching and antigen engineering can boost integrity and thermal stability of vaccine antigens, and lead to improved serum immune responses.

## Figures and Tables

**Table 1:** Physicochemical characterization of CsbD-based vaccine antigens.

Antigens *	Purity (%)	Endotoxin (EU/mg)	Solubility ^¶^ (mg/mL)	SE-HPLC (% AUC)	Secondary Structure ^†^	Melting Temperature (°C) ^†^
dscCsbD_WS6788_ (resolubilized)	97	231	NA	NA	β-sheets	NA
dscCsbD_WS6788_ (soluble)	100	15	≥8.0	95	β-sheets	71
dscCsbD_LSN139_	100	22	≥8.0	98	β-sheets	71
dscCsbD_LSN139_(P218A)	100	279	≥8.0	89	β-sheets	70
dscCsbD_LSN139_(G3)	100	19	≥8.0	97	β-sheets	73
dscCsbD_LSN139_(P218A/G3)	100	261	≥8.0	88	β-sheets	73

* Antigens were purified from soluble fractions if not specified. ^¶^ Solubility was measured after reconstitution of lyophilate. ^†^ Secondary structure and melting temperature were determined by circular dichroism spectroscopy.

## Data Availability

Data is included within the article and Supplementary Material.

## Supplementary Materials

The supplementary materials Figure S1 is available online at www.mdpi.com/article/10.3390/microorganisms9081646/s1.

## Disclaimer

All studies were reviewed and approved by the NMRC Institutional Animal Care and Use Committee and performed in compliance with all applicable Federal regulations governing the protection of animals in research. The views expressed in this article reflect the results of research conducted by the authors and do not necessarily reflect the official policy or position of the Department of the Navy, Department of Defense, nor the U.S. Government. I.V.E. and S.J.S. served as military service members over the course of this work. This work was prepared as part of their official duties. Title 17 USC. §105 provides that ‘Copyright protection under this title is not available for any work of the United States Government.’ Title 17 U.S.C. §101 defines a U.S. Government work as a work prepared by a military service member or employee of the U.S. Government as part of that person’s official duties.
