# Supplementary material for: Development and Comparison of a Panel of Modified CS17 Fimbrial Tip Adhesin Proteins as Components for an Adhesin-Based Vaccine against Enterotoxigenic Escherichia coli
Source: Microorganisms. 2021 Jul 31;9(8):1646. doi: 10.3390/microorganisms9081646 (PMC8401227; doi:10.3390/microorganisms9081646)

Figure S1. Circular dichroism spectra of $dscCsbD_{WS6788}$ (resolubilized) and $dscCsbD_{WS6788}$ (soluble).

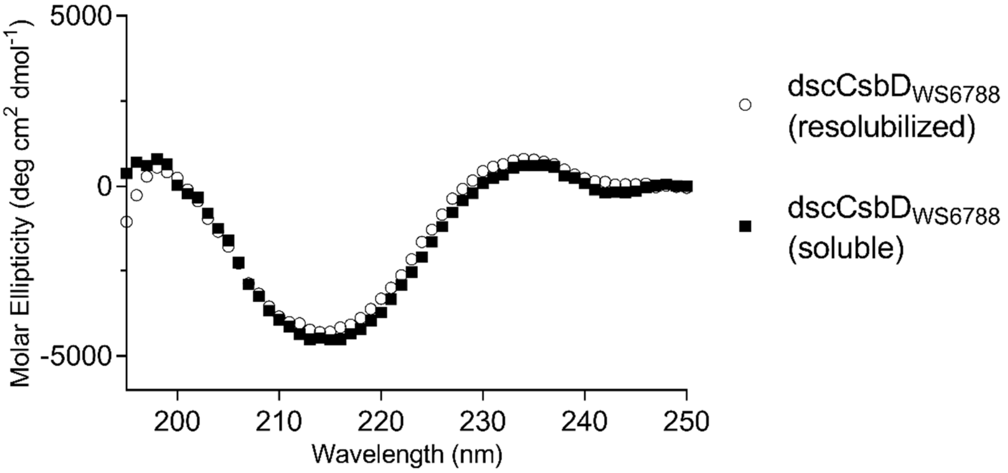

Supplement: Supplementary file 1 [file microorganisms-09-01646-s001.zip › microorganisms-1260201-supplementary.pdf]
